# Mapping the Conflict Between Oral Health and Patient Autonomy in Dentistry: A Unified Qualitative-Quantitative Study

**DOI:** 10.1016/j.identj.2025.104012

**Published:** 2025-11-06

**Authors:** Szilárd Dávid Kovács, Anna Jeney, Szilvia Zörgő

**Affiliations:** a Institute of Behavioural Sciences, Faculty of Medicine, Semmelweis University, Budapest, Hungary; b Department of Anthropology, College of Social Sciences, Seoul National University, Seoul, Republic of Korea; c Department of Society Studies, Faculty of Arts and Social Sciences, Maastricht University, Maastricht, The Netherlands

**Keywords:** Dental ethics, Beneficence, Dental esthetics, Paternalism, Personal Autonomy, Qualitative research

## Abstract

**Introduction and aims:**

Patients’ interests encompass both medical indication and their autonomous requests; however, these two aspects often conflict. We aimed to map this ethical dilemma considering dentists’ and patients’ perspectives.

**Methods:**

We included patient and dentist subsamples, applying quotas for sex and leadership experience among dentists, and quotas for sex and age among patients. We conducted semi-structured interviews, developed codes using prior theory, and inspected the coded dataset using Epistemic Network Analysis, a method that visualizes code co-occurrence patterns.

**Results:**

Dentists’ narratives indicated a preference for performing esthetic procedures that also had a medical indication; albeit, they encouraged patients to make treatment decisions by informing adequately. Patients often justified esthetic procedures by associating esthetics with health, while in other cases, they based decisions on prior experiences and perceived comfort.

**Conclusion:**

Ethical theory adopted from literature was insufficient to fully capture complexities, particularly in-patient narratives. These findings indicate a need for novel ethical approaches that better reflect patients’ reasoning.

**Clinical relevance:**

If individual dentists or higher-level stakeholders advocate for change, they must account for patient’s subjective perspectives and lived experiences.

## Introduction

Modern medicine recognizes that patients’ interests include both their medical necessities and making informed decisions regarding their body and health. Beauchamp and Childress regard principles related to medical necessity—namely beneficence and nonmaleficence—and the principle related to the patient’s perspective, respect for patient autonomy, as prima facie principles that should be fulfilled to the greatest extent possible, without ranking one above the other.^1^ Respect for patient autonomy was incorporated into the Declaration of Geneva in 2017, marking a shift away from the paternalistic approach historically represented in the Hippocratic Oath.^2^ In Bester’s philosophy, medical indication and patient autonomy not only coexist but actively interact, as e.g., patients’ informed decisions can guide the choice between beneficial treatment alternatives.^3^ However, not all cases are ideal, as a patient's autonomous decisions may sometimes be harmful for their health. Wilkinson provides examples in his paper of situations where a patient’s wish does not align with their best interest, such as the refusal of vaccines based on the belief of microchips hidden within, or rejecting a necessary cesarean section due to a belief in the superiority of “free birth.”^4^ In these cases, Wilkinson argues that since one’s moral values may undergo radical changes over time, and these shifts are not foreseeable, the ethical decision is not to grant wishes that may cause harm.^4^ Although the previously mentioned cases may appear as harmful, Kovács highlights that the distinction between harmful and beneficial patient requests is not always straightforward, revealing inconsistencies in bioethicists’ views.^5^ In Kovács’s example bioethicists generally support gender-affirming surgery, viewing its psychosocial benefits to outweigh the physical harm of rendering the patient infertile.^5^ At the same time, they commonly oppose the amputation of a healthy limb in patients with body integrity identity disorder, prioritizing the avoidance of physical harm over the intervention's psychosocial benefits.^5^

When examining the ethical challenges associated with patient autonomy, the field of dentistry, which specializes in the care of the craniofacial complex, is particularly susceptible to ethical considerations in treatment planning. This significance arises from the craniofacial complex’s fundamental role in an individual’s self-perception and social interactions. Beyond its physiological functions—sensory perception, taste, touch, respiration, yawning, mastication, salivation, and swallowing—the craniofacial complex also serves critical psychosocial functions. These encompass facial expressions, orofacial esthetics, intimate and social interactions such as kissing and speaking, as well as the shared experience of communal meals.^6^ Ethical challenges related to patient autonomy in dental care frequently arise in cases involving the refusal of medically indicated treatments or preventive care, requests for non-indicated tooth extractions, and the performance of esthetic procedures.^7^, ^8^, ^9^, ^10^, ^11^

In a previous scoping review on ethical challenges of patient autonomy in dentistry, we revealed that only a limited body of literature addressing this topic is available.^11^ Moreover, while most publications appeared in dental journals, none were found in bioethics journals (with our query terms and inclusion criteria). This perspective may explain why the emphasis in literature was placed on dentists' duty to provide care based on professional standards, scientific evidence, and legal norms.^11^ Another similar review and theoretical evaluation of literature on wish-fulfilling medicine and dentistry by Witter et al. examined cases in which patients perceive they have esthetic deviations from a normative standard and seek correction through cosmetic, orthodontic, or prosthetic interventions.^12^ The study scrutinizes dental cases through legal implications, and describes generally applicable arguments, such as the notion that if social pressure influences a person’s wishes, their autonomy may be compromised.^12^ A theoretical bioethical framework adapting Beauchamp’s and Childress’s principles to dental practice called the Central Practice Values was developed by Ozar, Sokol, and Patthoff to guide ethical decision-making by examining which goals of dentistry are in conflict.^13^^,^^14^ However, unlike Beauchamp and Childress, it establishes a hierarchy, prioritizing the patient’s life, general health, and oral health above all other values; in its most recent form, these are patient autonomy, dentists’ preferred practice patterns, esthetic value, and efficient resource use.^1^^,^^14^ Nonetheless, as the framework was developed from within the dental profession, it reflects the normative commitments of dental practitioners; conversely, as Rule and Veatch note, the public might rank values differently than practitioners, and there may not be a consensus among practitioners in the right hierarchy of ethical values.^15^

The objective of our study was to map the ethical dilemma that arises when a patient’s oral health and autonomy are in conflict during dental care to contribute to future theoretical studies developing ethical guidelines for dentistry. In light of this objective, our research question was: How do dentists and patients prioritize and connect the Central Practice Values proposed by Ozar, Sokol, and Patthoff when a patient’s request conflicts with medical necessity, and what additional values manifest in their respective narratives?^14^

## Materials and methods

We employed non-proportional quota sampling to sample from two populations: dentists and patients living in Hungary. A sex quota was applied for both subsamples based on Gilligan's theory, which describes differences between “masculine” and “feminine” approaches to resolving ethical dilemmas.^16^ For the dentist subsample, a professional experience quota involving leadership experience in a governmental or non-governmental professional organization, university clinic, or private clinic was employed. Governmental professional organizations consist of the roles of Dental Officer, Associate Leaders in Public Care, the Dental Section of the Hungarian Medical Chamber, and the Department of Dental and Oral Diseases of the Professional Medical Board. In the patient subsample, we applied an age quota, as previous literature has reported differences in satisfaction with oral esthetics based on age.^17^^,^^18^ We included dentists with accredited specialty training and at least 10 years of professional experience involving both public and private sectors in Hungary. Patients were included if they faced a conflict between oral health and autonomy within the past year, while those under 18 or with dentistry-related healthcare qualifications were excluded. A patient's request was considered inconsistent with medical indication if they refused treatment despite a recommended treatment plan (e.g., refusing extraction of a tooth if it does not cause symptoms, or refusing fabrication of dental prostheses in partial edentulism) or if intervention was sought without a corresponding clinical diagnosis (e.g., for purely esthetic purposes). Sample sizes were determined by the researchers via theoretical saturation and the fulfillment of the quotas.

We recruited dentists using their publicly available contact information and employing snowball sampling to reach additional dentists, as well as patients eligible for the study after the completion of their treatment. Data was collected between March 2023 and November 2024 with a self-developed sociodemographic survey and semi-structured interviews. Two distinct interview guides were employed for the two subsamples; however, both covered the following key topics: (1) ranking and reflecting on the Central Practice Values;^14^ (2) personal experience with the researched ethical dilemma; (3) analysis of a case by Rule and Veatch, in which the dentist of a fictive patient proposes a comprehensive treatment plan to preserve all teeth, while the patient prefers the extraction of all compromised teeth;^15^ (4) evaluating esthetic dental procedures (5) patients’ trust in dentists and attitude of seeking treatment from dental technicians. Our data collection tools are accessible at: https://osf.io/wk2yp. The interviews were sound-recorded and anonymized during verbatim transcription.

Two raters developed codes in an iterative process. They conducted guided-inductive coding sentence-by-sentence on 10% of the dataset via the Interface for Reproducible Open Coding Kit (iROCK)^1^ utilizing the Central Practice Values and developing additional codes as necessary.^14^ Table 1 presents the codebook employed for the initial coding process, comprising the Central Practice Values, a summary of the definitions provided by the authors, along with an additional code for instances that did not align with these values but were nonetheless relevant to addressing the research question.^14^ Subsequently, the raters triangulated their codes and formulated a tentative codebook, supplementing code labels and definitions with examples from the data. This codebook was tested deductively on an additional 10% of the data, and subsequently refined through another round of triangulation. After further testing and triangulation, a final codebook was established, presented in Table 2.

**Table 1:** Codebook utilized in the first step of code development, compromising the central practice values and an “Other” code for further ethical considerations.

Code label	Definition
Patient’s life and general health	Health considerations beyond oral health
Patient’s oral health	Appropriate and pain-free function of the oral cavity and surrounding tissues
Patient autonomy	Treatment decisions made based on patient’s own beliefs, goals, and values
Dentist’s preferred patterns of practice	Dentist’s preference of equipment, medication, materials, setting, treatment strategy; Decisions based on the dentist’s habits, experience, skills, and philosophy (e.g., to extract teeth as a last resort)
Esthetic values	Patient’s esthetic goals guided by the dentist’s knowledge of prevailing esthetic standards in society
Efficiency in the use of professional resources	Allocating resources (dental expertise, dentist’s capacity, physical resources) with the aim of maintaining accessibility to oral care for other patients and the larger society
Other	Further aspects related to the dilemma between patient autonomy and oral health

**Table 2:** Final codebook containing code labels, their definitions, and an illustrative example.

Code label	Definition	Example
Beneficence	Desire to fulfil “what is in best interest” of the patient; Desire to preserve the patient’s health or bodily integrity	Patient entrusting dentist with the treatment plan that serves oral health
Patient autonomy	Evaluating the patient’s own beliefs, goals and values; Including right to choose the dentist based on personal preference	Dentist allowing the patient to choose a treatment alternative
Professional autonomy	Considering the dentist’s personal set of values, practice patterns; The dentist’s personal responsibilities; The right to reject treating a patient	Dentist rejecting patient request due to personal beliefs
Laws and rules	Choosing a course of action in accordance with the laws governing dental practice; Including reference to quackery	Dentist outlining possible treatment options that are legally feasible
Feasibility	Weighing the constraints of a desired treatment outcome; What is realistic; What is modern or outdated	Patient desiring interventions that are available in “more developed countries than Hungary”
Medical indication	Evaluation of a treatment plan based on function or physiology; Changes in function post treatment	Patient requesting a durable solution
Informing	Decision made based on access to information; Informing patients without the intent to convince them; Patient desire to be fully informed	Patient rejecting a treatment plan due to lack of information
Patient comfort	Minimizing the number of sessions, time spent at the dental office, fear (of the treatment, not of dentists in general), or inconvenience of the intervention	Patient requesting an intervention that is performed fast and pain-free
Finance	Decision based on financial considerations	Dentist claiming a treatment alternative is often not affordable for patients
Plurality	The acknowledgement of multiple moral systems; Including: Dentist and patient viewpoints, cultural differences	Dentist claiming that one patient requests one option, while the other patient requests a different option, when both are valid
Needs	Ethicality of an intervention is based on a specific circumstance	Dentist claiming that whitening teeth is ethical before a bride’s wedding to make that day special
Health attitude	Evaluating the feasibility of a treatment based on oral hygiene or regular check-ups	Dentist elaborating that they will advise different treatment for a patient who smokes, especially if the tooth causing symptoms has an unfavourable prognosis
Prestige	Confidence towards the dentist or medicine as a whole; Public perception of dentistry and medicine	Patient claiming that a “real” dentist would not want to extract their teeth
Triangulation	Weighing others’ opinions	Patient seeking information on social media
Personal experience	Decisions informed by prior events in which participants were either directly involved or had observed others	Patient not trusting dentists in public healthcare due to childhood memories
Esthetics	Esthetic goal, in which esthetics can be defined by the individual or by society	Patient rejecting amalgam fillings due to their metallic colour
Minimally invasive	Opting for the alternative that involves less damage to tissues or structures	Dentist claiming that an esthetic intervention is ethical, if it does not require tooth preparation
Well-being	Considering the impact on general health (biopsychosocial well-being); Aspects beyond medical indication	Dentist arguing for the fabrication of an esthetic prosthesis due to its positive psychological effects

The dataset was segmented according to sentences using the R package {rock}.^2^ Subsequently, coding was performed utilizing iROCK, and the data was structured into a tabular format with the R package, where rows represented lines of data, while columns contained attributes, assigned codes, and data. For a comprehensive description of data segmentation, please see:^19^

This structured dataset was then utilized for Epistemic Network Analysis (ENA)^3^ by constructing a pairwise code co-occurrence matrix of segments defined by moving windows consisting of two lines of data and aggregated for each participant and the subsamples. The resulting vectors of co-occurring codes were processed using dimensional reduction techniques, means rotation and singular value decomposition, to project them into a two-dimensional space. In this representation, nodes correspond to codes, edge weights reflect the relative frequency of code co-occurrences, and node positioning captures similarity in code co-occurrence patterns within the entire dataset. For a comprehensive description of ENA, please see:^19^, ^20^, ^21^, ^22^

In the following sections, code labels appear capitalized in italics, while narratives—originally in Hungarian and translated by the first author—are presented in quotation marks.

## Results

Our study included 14 dentists and 10 patients; Table 3 summarizes their attributes. To prevent dentists from becoming identifiable, we intentionally did not align attributes in the table such as birth year with the leadership role. For the sake of consistency—and as our results describe group means, not individual cases—we applied the same approach to patients.

**Table 3:** Attributes of participants.

Subsample	Attribute	Categories within an attribute	Number of participants (including overlaps)
Dentists	Sex assigned at birth	Male	N = 8
		Female	N = 6
	Specialty training	Prosthodontics or Prosthodontics and conservative dentistry	N = 11
		Dental and oral diseases	N = 7
		Oral implantology	N = 7
		Dentoalveolar surgery	N = 3
		Periodontology	N = 1
		Orthodontics	N = 1
	Leadership experience	University clinic	N = 6
		Private clinic overseeing subordinate dentists	N = 5
		Professional organization	N = 7
		No leadership experience	N = 4
Patients	Sex assigned at birth	Male	N = 4
		Female	N = 6
	Age group	Below 35 years old	N = 4
		35 to 65 years old	N = 4
		Above 65 years old	N = 2
	Education level	Tertiary	N = 8
		Secondary	N = 1
		Primary	N = 1
	Reported financial status	Monthly income sufficient to frequently save money	N = 4
		Monthly income sufficient to occasionally save money	N = 5
		Monthly income usually not sufficient to save money	N = 0
		Did not answer	N = 1
	Decisions contradicting medical indication	Refusal of intervention	N = 7
		Request for intervention	N = 3

Dentist narratives frequently drew on the Central Practice Values outlined by Ozar, Sokol, and Patthoff;^14^ however, the raters reached consensus on certain modifications to the original definitions to more accurately capture the concepts articulated by the participants. The paramount value in the hierarchy, the patient’s life and general health was represented by the *Well-being* code. While we did not explicitly include life in our definition—since it did not emerge in the narratives—we considered that a life-threatening event would be coded as one that harms well-being. Though the exact definition is not defined in the Central Practice Values, we adopted a holistic definition, aligning with the World Health Organization’s concept of health as biopsychosocial well-being.^14^^,^^23^

The patient’s oral health was adopted from the Central Practice Values as *Medical indication*, which referred to proper function, the definition used both in the Central Practice Values and by the Fédération Dentaire Internationale.^14^^,^^24^ *Patient autonomy* was also directly adopted from the Central Practice Values, aligning with the concept described by Beauchamp and Childress.^1^^,^^14^ However, we specifically emphasized the patient’s right to choose their dentist in our definition, as this emerged frequently in the narratives. Additionally, we introduced *Informing* as a separate code, which even though is related to the patient’s autonomy, it does not inherently ensure that the patient’s values are taken into account. The definition of esthetic values was modified in *Esthetics*, as the Central Practice Values describes this value as the dentist’s interpretation of societal norms,^14^ and the narratives did not describe a conflict between individual and societal, or between dentist and patient perceptions of esthetics.

Two values described in the Central Practice Values were not included in our final codebook, as they did not appear in the narratives, the dentist’s preferred patterns of practice and efficiency in the use of professional resources.^14^ Albeit, the dentists’ perspectives and practice patterns were incorporated in *Professional autonomy*, the term in this context does not primarily refer to clinical choices such as the selection of materials. Instead, it predominantly captures a concept positioned in contrast to patient autonomy—namely, the dentist’s rights and responsibilities in clinical decision-making. Additionally, while *Finance* may relate to resource use, it was observed in the narratives at the individual dentist-patient interaction level, rather than in the broader societal context described in the Central Practice Values.^14^ Beyond these adaptations, we introduced several additional codes derived from the *Other* code, as depicted in Table 2.

**Fig. 1:**
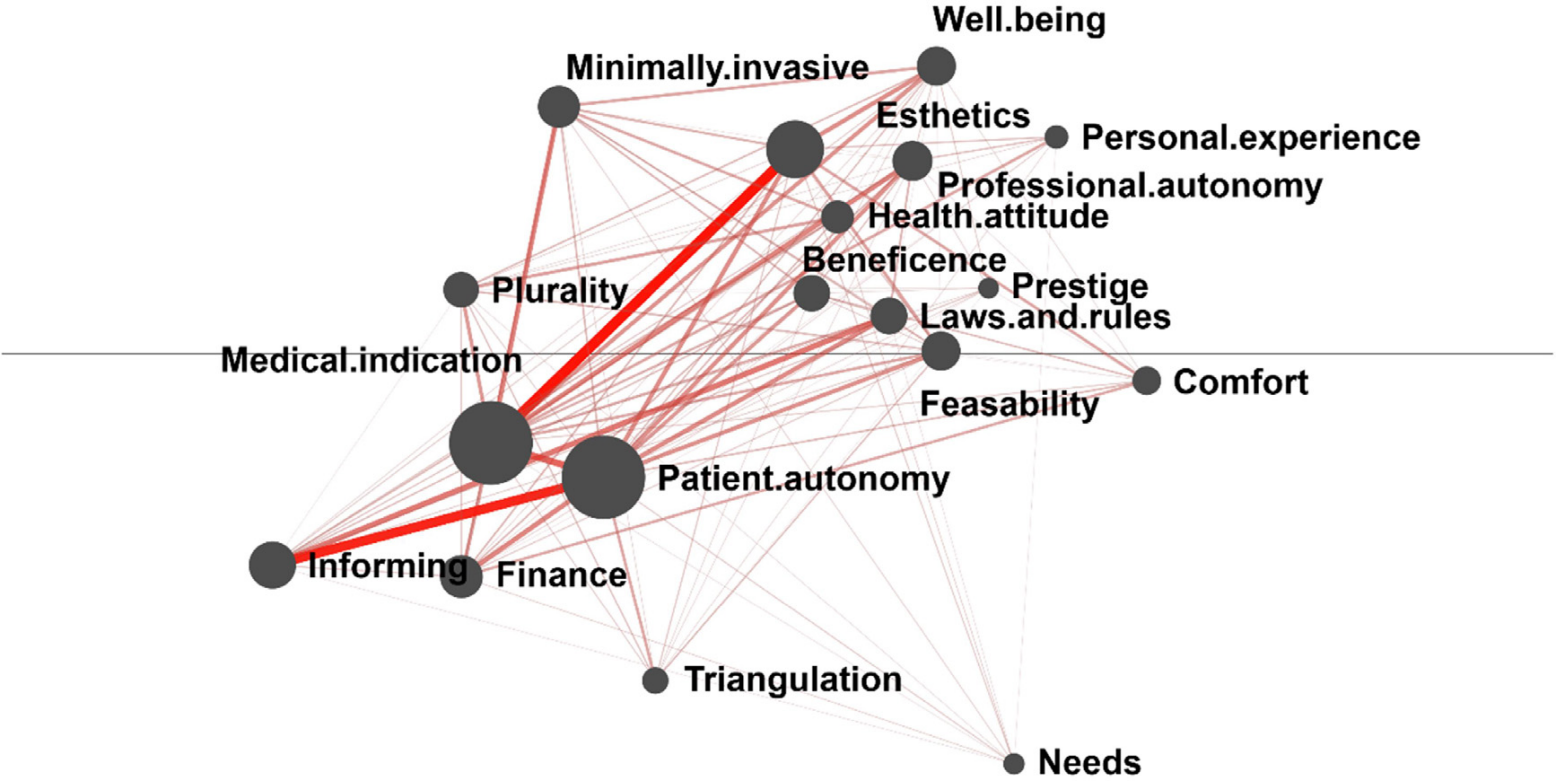
Mean epistemic network of the dentist subsample. Codes are represented by the nodes of the network (black circles). The size of each node reflects the relative frequency of the code’s co-occurrences with other codes. The relative frequency of co-occurrence between specific pairs of codes is indicated by the thickness and saturation of the edges (lines) between nodes.

Figure 1 displays the mean epistemic network of dentists. The network was densely connected, with the strongest associations exhibited by codes *Medical indication, Patient autonomy*, and *Esthetics*; the highest frequency of co-occurrence was observed between *Medical indication* and *Esthetics* and between *Patient autonomy* and *Informing*. The connection between *Medical indication* and *Esthetics* is well-represented by the dentist statement: “The request to remove calculus is quite frequent, but from a patient’s perspective, it is a cosmetic issue. (…) For them, it is primarily an esthetic concern, but we understand that the primary benefit is not merely cosmetic.” Similarly, another dentist described the necessity of replacing dental bridges as “We know our body changes, the marginal fit [of the crown] may become insufficient, maybe it is no longer sufficiently esthetic.” These findings suggest that while dentists may not prioritize esthetic outcomes as a primary professional concern, based on their clinical experience, dentists infer that patients’ motivation to seek dental treatment is often driven by esthetic concerns. Thus, dentists tend to prefer interventions that align both with their professional assessment of oral health benefits and the patient’s perceived esthetic preferences to encourage patient compliance with beneficial treatments, rather than performing merely esthetic interventions.

The co-occurrence of *Patient autonomy* and *Informing* highlights a different perspective. One dentist stated: “If they are fully informed, they have the right to make decisions regarding their oral health.” Another participant remarked “I do not begin any treatment unless I have informed the patient and they have provided consent.” These statements indicate that dentists recognize informing as a mechanism that enables patients to share responsibility in managing their oral health. Another code associated with patient responsibility, *Health attitude,* exhibited only weak connections to *Patient autonomy*, suggesting that within dentists’ narratives, a patient’s responsibility for self-care in their lifestyle is conceptualized as distinct from their right and responsibility for their health when making decisions about clinical interventions.

**Fig. 2:**
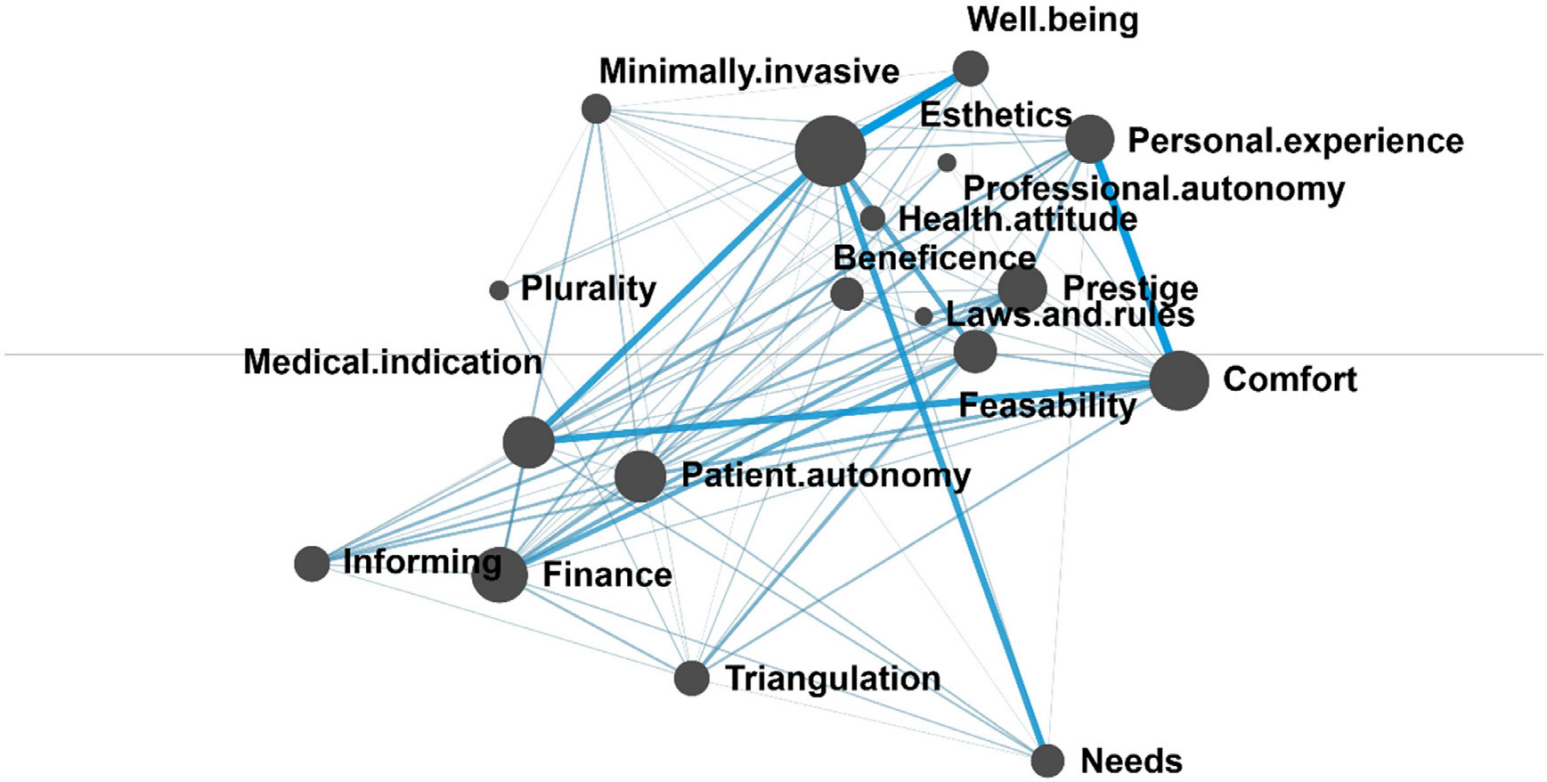
Mean epistemic network of the patient subsample. Codes are represented by the nodes of the network (black circles). The size of each node reflects the relative frequency of the code’s co-occurrences with other codes. The relative frequency of co-occurrence between specific pairs of codes is indicated by the thickness and saturation of the edges (lines) between nodes.

The mean network of patients depicted in Figure 2 was also densely connected, and *Medical indication* and *Patient autonomy* maintained a high frequency of co-occurrence in this network as well. Conversely, several significant codes were identified that are not included in the Central Practice Values framework, such as *Finance, Personal experience, Comfort, Feasibility,* and *Prestige* (13). The strongest co-occurrences were observed between *Esthetics* and *Well-being* and between *Personal Experience* and *Comfort*.

An illustrative example of the connection between *Esthetics* and *Well-being* is the patient remark: “These two are intertwined. If something is healthy in the first place, then it looks good, and it has to look good.” Another patient emphasized the psychosocial benefits of an esthetic intervention as follows, “The price was 100-120 thousand forints, which is not a small amount, but compared to other cosmetic procedures, it’s not so bad (…) the real advantage for me is the confidence boost, that I can smile freely.” This statement suggests that while dentists primarily tended to integrate their perception of medical indication with patients’ esthetic desires, patients conceptualized health in two additional ways: first, as “looking healthy”, and second, as a facet of psychosocial function and overall health.

The co-occurrence of *Personal experience* and *Comfort* can be exemplified by a patient explaining their motivation for requesting a crown preparation also involving neighboring teeth for solely esthetic purposes: “I was afraid that the filling in my tooth would fall out, it has happened before, and it was really unpleasant. I couldn’t get a dental appointment immediately, and my tooth fragment was loose.” Another patient, describing their concerns about trust in dentists stated: “On the third occasion, I thought to myself that I would tell [the patient admission coordinator] I’m not disabled, I didn’t come for an examination because of any brain issue, so the they should talk to me like a normal person (…) so I don’t like going back to a place where I am spoken to in a condescending manner.” These examples illustrate that patients draw upon past experiences involving discomfort or inconvenience in their decision-making. In the first example, a prior dental treatment was associated with unpleasant outcomes, leading the patient to seek a different restorative method to avoid a similar experience. In the second example, previous negative experiences with communication contributed to the avoidance of beneficial treatment.

## Discussion

A general challenge in bridging theory and empirical research is that theoretical frameworks are usually not directly suited for empirical application. In our study, we addressed this by operationalizing the theory of the Central Practice Values through adapting the definitions provided by the authors.^14^ Rather than ranking these values within a hierarchy, as initially proposed, we focused on examining the relationships between them and interpreting the meanings of these connections within narratives. This form of operationalization was intended to enable a more nuanced understanding of how these values are negotiated in everyday clinical practice. Our findings revealed that dentists hold a perspective similar to that observed among general practitioners and plastic surgeons in a study by Asscher et al.^25^ fulfilling patient wishes can facilitate the delivery of medically necessary treatment. However, in dentistry, this alignment is not primarily about maintaining patient trust, but rather about offering interventions that simultaneously address both medical and patient-driven goals. Furthermore, we found that the act of informing patients is employed by dentists as a tool to support patient autonomy, not merely to fulfil an ethical obligation, but to empower patients by transferring responsibility to them.

As the Central Practice Values represent a particular perspective rooted in dentistry, and indeed only one set of several possible dental perspectives, we modified the definitions and introduced new concepts.^14^^,^^15^ For instance, life was rarely mentioned by participants, and in discussions of esthetics, it was often unclear whether the reference point was dentist, patient or societal expectations. Furthermore, the substantial number of additional codes we introduced highlights the incompleteness of the original theory and suggests that many other aspects influence clinical reasoning. Our findings show that dentists’ reasoning aligned more closely with the concepts present in the Central Practice Values and with additional concepts commonly discussed in bioethics, such as financial considerations, legal obligations, and patient information.^14^ In contrast, among patients, for example a strong co-occurrence was observed between *Comfort* and *Experience*, indicating a logic that may be more aligned with case-driven approaches than with principlism, as this theme draws on analogies from own experiences and prioritizes an individual sense of comfort, forming decisions based on personal relevance.^26^ This also suggests that patients do not explicitly articulate expectations, such as their right to be informed or to make autonomous decisions. If such rights are to be upheld in practice, it becomes the responsibility of the dentist to actively facilitate them. Encouragingly, our findings indicate that many dentists are indeed willing to do so.

## Limitations

The main limitation of ENA is its usage of code pairs, which constrains its ability to capture the potential significance of codes in isolation or more complex code constellations beyond dyads. A limitation of the transferability of findings relates to the Hungarian context, as all conservative dental treatments are covered in the public healthcare system, which may account for the relatively low frequency of financial concerns expressed by patients. Furthermore, the study specifically addressed decisions made at the patient–dentist level, whereas some decisions relevant to the ethical dilemma occur at higher levels, as research on dentists’ decision-making in other topics has differentiated according to the level at which decisions are made.^27^ Finally, while our analysis focused on subsamples, this group-based approach may have obscured individual nuances that could have emerged through a more granular, case-level analysis.

## Conclusion

Dentists’ ethical reasoning reflects principles more common in bioethics, in contrast to patients relying on subjective, experience-based insights. If dentists aim to challenge such perspectives, e.g., the overvaluation of esthetic appearance, they must ensure this does not discourage dental visits, which are often motivated by these concerns. Furthermore, as principlism aligns less with patients’ thinking, future ethical guidelines need to expand the scope of ethical principles or consider alternative theoretical frameworks.

## Funding

Supported by the EKÖP-2024-2.1.1-EKÖP-2024-00004 University Research Scholarship Programmed of the Ministry for Culture and Innovation from the source of the National Research, Development and Innovation Fund

## Ethics approval

The project gained approval from the Medical Research Council’s Scientific Research Ethics Committee (Egészségügyi Tudományos Tanács Tudományos és Kutatásetikai Bizottság). Reference number for the ethics approval: BMEÜ/3278- 1 /2022/EKU.

## Declaration of generative AI in scientific writing

The authors have not used AI for the study.

## Author contributions

SK was responsible for conceptualization, data curation, formal analysis, funding acquisition, investigation, project administration, resources, software, visualization and writing (original draft). AJ was responsible for data curation and formal analysis. SZ was responsible for conceptualization, methodology, project administration, supervision, validation, writing (reviewing and editing).

## Conflict of interest

The authors have no competing interests to declare.

## Footnotes

1 https://i.rock.science

2 https://rock.science

3 https://www.epistemicnetwork.org
